# Noise induced hearing loss impairs spatial learning/memory and hippocampal neurogenesis in mice

**DOI:** 10.1038/srep20374

**Published:** 2016-02-04

**Authors:** Lijie Liu, Pei Shen, Tingting He, Ying Chang, Lijuan Shi, Shan Tao, Xiaowei Li, Qingying Xun, Xiaojing Guo, Zhiping Yu, Jian Wang

**Affiliations:** 1 Department of Physiology, Medical College of Southeast University, Nanjing China; 2 Children’s medical center, the Second Affiliated Hospital of Nanjing Medical University, Nanjing, China; 3 School of Human Communication Disorders, Dalhousie University, Halifax, Canada

## Abstract

Hearing loss has been associated with cognitive decline in the elderly and is considered to be an independent risk factor for dementia. One of the most common causes for acquired sensorineural hearing loss is exposure to excessive noise, which has been found to impair learning ability and cognitive performance in human subjects and animal models. Noise exposure has also been found to depress neurogenesis in the hippocampus. However, the effect is mainly attributed to the oxidant stress of noise on the cognitive brain. In the present study, young adult CBA/CAJ mice (between 1.5 and 2 months of age) were briefly exposed a high sound level to produce moderate-to-severe hearing loss. In both the blood and hippocampus, only transient oxidative stress was observed after noise exposure. However, a deficit in spatial learning/memory was revealed 3 months after noise exposure. Moreover, the deficit was correlated with the degree of hearing loss and was associated with a decrease in neurogenesis in the hippocampus. We believe that the observed effects were likely due to hearing loss rather than the initial oxidant stress, which only lasted for a short period of time.

Hearing loss is one of the most common neurological disorders, affecting 10% of the general population^1^ and more than 40% of those over 60 years of age^2^,^3^. Noise exposure is one of the major causes of acquired sensorineural hearing loss in adults^4^. However, the damaging effects of noise are not limited to the auditory system but rather extend to many other systems^5^. Recent studies have discussed the noise-related impairment of learning ability and cognitive performance^6^,^7^,^8^,^9^. Soldiers who were exposed to excessive noise levels, including explosions and blast waves, experienced severe noise-induced hearing loss (NIHL) and tinnitus^10^,^11^ as well as cognitive deficits and memory impairment^12^.

The mechanisms underlying the decline of cognitive functions after noise exposure are not entirely clear. Animal studies have suggested that noise exposure is likely to impair cognitive functions through two different but closely related approaches. One is due to the oxidative reaction initiated by noise exposure. Many studies have shown that increased oxidative stress is the cause of neuronal degeneration in many auditory nuclei as well as the brain regions critical for cognitive functions^6^,^7^,^13^,^14^. Another potential effect of noise exposure is a change in the auditory input to the cognitive brain after NIHL. Overall, this effect has largely been ignored in studies of the impact of noise on cognitive function and neurogenesis; however, its existence is supported by the connection between the auditory brain and cognitive brain^15^, the hippocampal degeneration and deterioration of spatial memory observed in CBA/CAJ mice with age-related hearing loss^16^, and the suppression of hippocampal neurogenesis observed in the rats long after unilateral NIHL^17^.

In the present study, CBA/CAJ mice were exposed to noise at a high level (123 dB SPL) but for a brief period of time (2 h) to produce permanent hearing loss. Cognitive performance and hippocampal neurogenesis were examined 3 months after noise exposure, long after the disappearance of oxidative stress. The long delay between the presence of oxidative stress and cognitive examination allowed for the investigation of the role of NIHL, rather than oxidative stress, in the decline of hippocampal learning/memory function and neurogenesis.

## Results

### Noise-induced hearing loss

**Figure 1:**
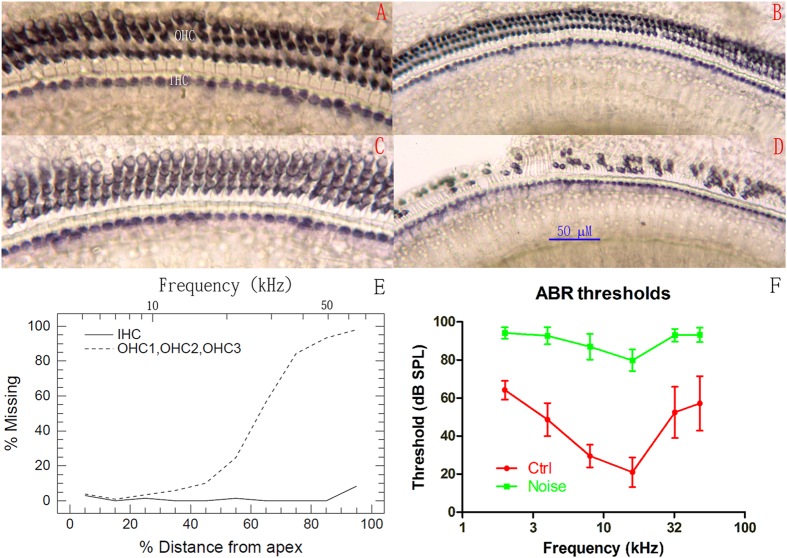
Noise-induced hair cell loss and ABR threshold shift measured 3 months after the noise exposure at 6–8 weeks of age. (**A**–**D**) Representative images of SDH staining of HCs from the two groups. (**A**,**B**) Apical and basal turns, respectively, in the control group; (**C**,**D**) Apical and basal turns, respectively, in the noise group. Massive OHC loss is clearly observed in (**D**). (**E**) Cochleogram of the noise-exposed animals showing the loss of OHCs concentrated in the basal half of the cochlea. (**F**) ABR thresholds. The differences between the two groups show that moderate-to-severe hearing loss was established in the noise group.

Figure 1A–D shows representative images of succinate dehydrogenase (SDH) staining on both outer and inner hair cells (OHCs and IHCs) from the two groups. Two images were presented for each group, one from the apical turn (Fig. 1A,C) and the other from the basal turn (Fig. 1B,D). In contrast to the spread of hearing loss on the ABR audiogram, it is clear that HC loss was limited to the basal half of the cochlea, mainly in OHCs. This is further demonstrated in the cochleogram of HC loss in Fig. 1E.

ABR audiograms were obtained at the age of 4.5–5 months and compared between the control group and the noise-exposed group to determine differences in hearing sensitivity. Figure 1F shows that the ABR thresholds of the noise group were much higher than those of the control group at every frequency tested. The frequency-averaged threshold was found to be 89.972 ± 2.907 dB SPL in the noise group, which was significantly higher than the value of 45.5 ± 6.995 dB SPL found for the control group (mean ± SD, n = 30; Student t test, t = 32.156, p < 0.001). The mean difference of 44.47 dB between the two groups suggests a moderate-to-severe degree of hearing loss in the noise group.

### Impact of NIHL on spatial learning and memory

**Figure 2:**
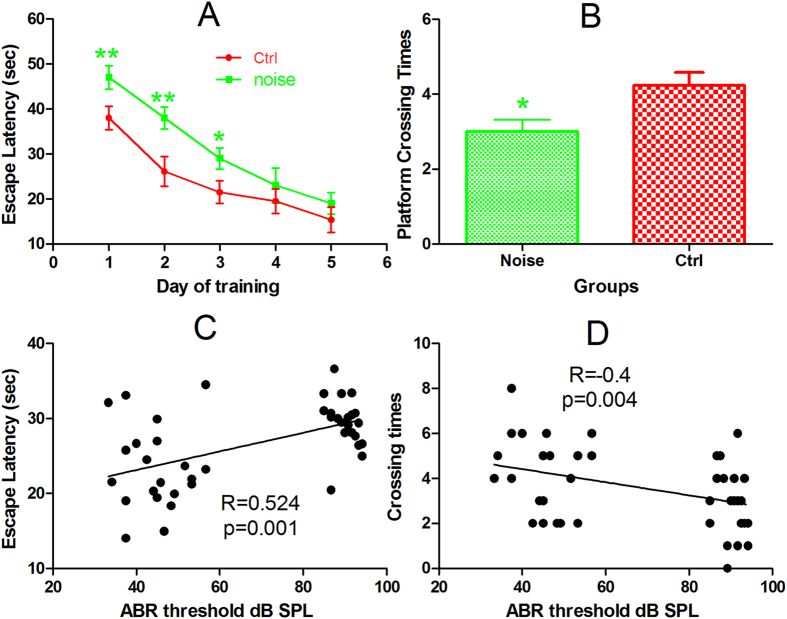
Effect of NIHL on MWM performance measured 3 months after the noise exposure at 6–8 weeks of age. (**A**) Change in escape latency with training days, (**B**) Platform crossing time in 60 second probe test. **p < 0.01, *p < 0.05. (**C**) Correlation between ABR threshold and escape latency, (**D**) Correlation between ABR threshold and platform crossing time. Inserted lines are the results of linear regressions.

Spatial learning ability and memory was tested in a Morris water maze (MWM). The first stage of this test involved a five-day training period in which the mice learned to find the hidden platform underwater (measured as escape latency) using spatial cues around the pool. Figure 2A compares the changes in escape latency during the training days between the two groups. The figure demonstrates slower learning in the noise group. A two-way ANOVA with the factors of noise exposure and days of training revealed a significant effect of noise exposure (F1 = 7.229, p < 0.008) on escape latency. Post-hoc pairwise comparisons (Tukey method) showed that the escape latency of the noise group was significantly longer than that of the control group on days 1, 2 and 3 of training (indicated by the asterisks in Fig. 2A). The second stage of the MWM was the spatial orientation test, in which the hidden platform was removed, and the number of crosses through the platform area within 60 seconds was counted as an index of the spatial memory of the platform. This number was 3.00 ± 0.32 in the noise group, which is significantly lower than the value of 4.23 ± 0.35 obtained in the control group (one tail Student *t* test, *t* = 2.946, n = 22 in each group, *p* < 0.05, Fig. 2B).

### Correlation between NIHL and MWM results

A Pearson correlation analysis was performed to verify whether the degree of NIHL is correlated to the outcome of MWM tests. A significant positive correlation was observed between the frequency-averaged ABR threshold and the averaged escape latency over the 5 days of training for each individual (Fig. 2C, r = 0.524, p = 0.001). In addition, there was a negative correlation between the frequency-averaged ABR threshold and the spatial memory manifested by the platform crossing times in the spatial orientation experiment (Fig. 2D, r = −0.4, p = 0.004).

### Oxidative Stress

**Figure 3:**
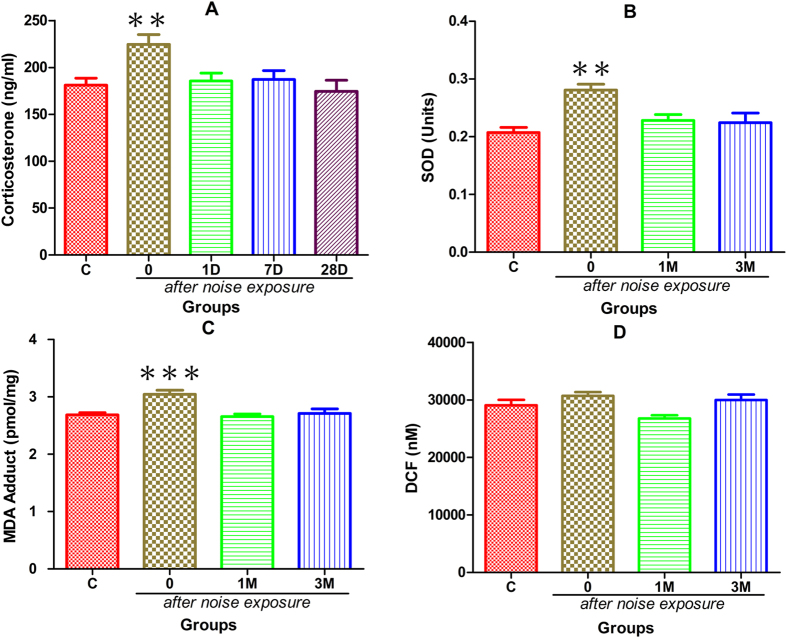
Effects of noise exposure on oxidative stress measured at different time points after noise exposure at 6–8 weeks of age. The time points of the evaluation are indicated in the figure (d: days, M: month after the noise exposure) (**A**) Plasma CORT hormone levels, (**B**) SOD activity, (**C**) MDA adduct content, (**D**) DCF level in hippocampus. The quantity of ROS/RNS was calculated based on the measure of DCF fluorescence (see methods section for details). (Mean ± S.E.M., n = 8, **p < 0.01, ***p < 0.001).

Figure 3A shows the change in serum corticosterone (CORT) level after noise exposure. A one-way ANOVA was performed and showed that the serum CORT level (224.44 ± 10.63 ng/ml) was significantly higher in the noise group than in the control group, as also shown by the post-hoc pairwise tests; however, this difference was only observed immediately after noise exposure, suggesting a transient effect of noise on CORT level. The changes in superoxide dismutase (SOD, Fig. 3B) and malondialdehyde (MDA, Fig. 3C) levels in the hippocampus were also transient (recovered within 1 month after noise exposure), and no significant change in ROS/RNS (Fig. 3D, measured by DCF fluorescence) was observed.

### Neurogenesis in the hippocampus

**Figure 4:**
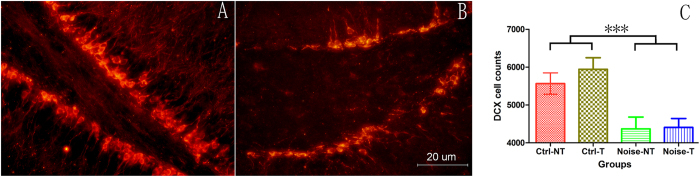
Comparison of DCX cells. (**A**) Representative images of the control group and (**B**) noise group. (**C**) Statistical comparison. ***p < 0.001 (between the noise and control groups).

**Figure 5:**
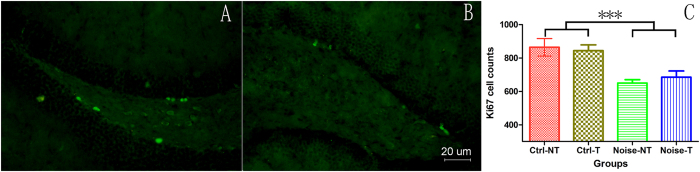
Comparison on Ki67 cells 3 months after noise exposure at 6–8 weeks of age. (**A**) Representative images of the control group and (**B**) noise group. (**C**) Statistical comparison. ***p < 0.001 (between the noise and control groups).

The impact of NIHL on neurogenesis was evaluated by counting newly generated hippocampal cells in the dentate gyrus (DG) region using antibodies against DCX (a marker specific to neurons newly generated within the last 2–3 weeks) and Ki67 (a marker of proliferation). The representative images for both DCX (Fig. 4A,B) and Ki67 staining (Fig. 5A,B) indicate a decrease in neurogenesis in the noise group. Figures 4C and 5C compare the average counts of DCX/Ki67 positive cells in the whole DG region in both groups. Two-way ANOVAs were performed for both DCX and Ki67 cell counts against the factors of noise and training in the MWM. A significant effect of noise was observed for both the DCX and Ki67 cell counts (F1 = 22.768, p < 0.001 for DCX cells and F1 = 23.228, p < 0.001 for Ki67 cell counts) but not for the factor of training (no difference between the trained and non-trained subjects in either the noise or control groups). The pooled number of DCX positive cells of the control group was 5756 ± 208, which was significantly higher than that of the noise group: 4387 ± 191 (t = 4.844, p < 0.0001). Similarly, the pooled numbers of Ki67 cells were 854 ± 30 and 668 ± 22 for the control and the noise groups, respectively, and the difference was statistically significant (t = 5.115, p < 0.0001).

## Discussion

In the present study, young adult CBA/CAJ mice were briefly exposed to noise, producing NIHL, which was verified to be of a moderate-severe degree by comparing the ABR threshold between the two groups at three months after noise exposure (Fig. 1E). Because the highest sound level in the ABR test is 90 dB SPL, the threshold over this level was assigned as 95 dB SPL. This ceiling effect was likely to cause an under-estimation of the threshold in the noise group. The effect of oxidative stress produced by the noise exposure was transient, as indicated by the changes in serum CORT level and the levels of three commonly used oxidative detection agents in the hippocampus (Fig. 3). The MWM test at this age showed that the animals in the noise group were slower in spatial learning and poorer in spatial memory (Fig. 2). Performance in the MWM appeared to be well correlated with hearing threshold (Fig. 2C,D). Furthermore, the NIHL appeared to decrease the level of neurogenesis in the hippocampus.

The adverse impact of noise on learning and memory and hippocampal neurogenesis has been reported by many previous studies^5^,^9^,^18^,^19^,^20^,^21^,^22^,^23^. Most of those previous studies focused on the oxidative stress induced by noise; thus, in those studies, the cognitive functions and changes in brain morphology and molecular content were observed shortly after or even during the period of noise exposure, when the oxidative stress response was strong. The possibility of NIHL, rather than noise-induced stress, having an effect on cognitive function has largely been ignored. In fact, in many previous studies, the noise level was so low that hearing loss, in terms of changes in hearing sensitivity, was not expected to occur and was therefore not even documented. For example, impaired learning and memory capabilities were found in mice after exposure to white noise at 80 dB SPL 2 hours per day for a 6-week period, and the deterioration in learning and memory caused by this noise exposure was attributed to the increased level of MDA and SOD detected in the inferior colliculus, auditory cortex and hippocampus^6^. Similar results were reported in other studies that investigated noise exposure at higher levels (100 dBA for 4 hours daily for 30 days)^24^.

While the role of noise-induced stress in cognitive impairment and neurogenesis is widely recognized, the independent effect of NIHL should also be considered, especially in cases of brief noise exposure. We think that the stress induced by the brief noise exposure in the present study is unlikely the reason for the deteriorated cognitive function and the depressed neurogenesis measured three months after the noise exposure. This argument is based upon several reasons. First of all, it has been recognized that adrenal glucocorticoid hormones are common pathway to the negative impact of stress on neurogenesis and related cognitive functions^25^,^26^,^27^. This connection has been demonstrated by (1) that cells in hippocampus express high level of glucocorticoid (GC) receptors^28^,^29^, which make them sensitive to GC mediated damage, (2) that administration of exogenous GCs decreases neurogenesis, and (3) that blockage of GCs prevents stress induced depression on neurogenesis^30^. Secondly, the impact of acute (brief) stresses of many different types on stress hormones is transient: it occurs in hours or days and quickly recovered after exposure to stressor^30^,^31^,^32^. The GC level is under restrictive regulation to which hippocampus neurons actively participate: the newly generated neurons here play a critical role in appropriate shut-off of the stress induced GC responses^33^,^34^. This is also true in the oxidative stress induced by brief noise exposure reported by others^35^,^36^ and in the present experiment. Thirdly, in most cases, long-lasting detectable changes in neurogenesis occur after prolonged and severe stress exposure^32^. Lastly, limited but significant data are available showing that the effect of transient noise exposure is transient. For example, spatial memory (similar to what we examined) was found to be deteriorated in a temporal pattern after impulse noise exposure at 165 dB SPL in rats: a totally recovery was seen within 24 hours after the noise^18^. In another study, a brief exposure to tone of 16 kHz at 110 dB SPL for 1 hour failed to show any changes in various behavioral tests for learning and memory^37^.

To further confirm the contribution of negative impact of hearing loss, the cognitive functions and neurogenesis should be evaluated by using conditional knockout mouse models in which hearing loss can be produced in adulthood with no or minimal stress. However, this does not devalue the noise model because NIHL impacts a larger population.

The possible impact of NIHL (rather than oxidative stress) on cognitive function is also supported by several lines of previous research data. Firstly, recent studies have demonstrated that hearing loss in general is an independent risk factor of dementia^38^,^39^,^40^,^41^; however, exactly how hearing loss promotes the development of dementia still has to be investigated. Secondly, a strong anatomical and functional connection exists between brain regions in terms of auditory and cognitive functions. The hippocampus receives auditory input through the lemniscal ascending pathway, which transmits acoustic stimuli from the inferior colliculus to the auditory cortex and then to the hippocampus^42^. Furthermore, the hippocampus projects indirectly to the auditory cortex^43^. The auditory association cortex has both direct and indirect pathways to the hippocampus and receives its’ indirect input. These connections enable the formation of long-term auditory memories and facilitate the processing of linguistic and musical input^15^. Thirdly, reduced auditory input after peripheral hearing loss has been reported to cause hippocampal degeneration and impaired memory function. For example, CBA/CAJ mice with age-related HL demonstrated hippocampal degeneration and had reduced spatial memory, as indicated by the results of the MWM test^16^. Even in cases where no hearing loss occurred, as indicated by the lack of change in hearing sensitivity (such as that reported in some previous studies that addressed the effect of noise on cognitive function), reduced auditory input from the peripheral hearing organ to the brain is expected based on recent studies of ‘hidden’ noise-induced hearing loss. Noise exposure producing no permanent threshold shift can largely damage the synapses between inner hair cells and the primary auditory neuron and therefore reduce the output of the cochlea to the brain^44^,^45^,^46^. Finally, the possible impact of HL on cognitive function is supported by the correlation of auditory threshold and the score of MWM performance.

A significant reduction in cell proliferation and neuronal generation was observed in the noise group, similar to that reported by other researchers^17^,^36^,^37^,^47^. Our study took the additional step of linking the decrease in hippocampal neurogenesis with the poorer performance in regard to spatial learning and memory during training in adult animals. However, we did not find any impact of training on the number of DCX or Ki67 cells in either the control or noise group. This result is inconsistent with the fact that the major effect of training is on the rescue of cells generated within one to two weeks prior^48^,^49^,^50^. For example, in rats, approximately 9,000 new cells are generated every day in the hippocampus^51^, and between one and two weeks after their generation, many of those cells die if they are not used in association with learning and memory^52^. Therefore, this period is considered critical for training in order to rescue those cells^53^,^54^,^55^. However, the DCX staining used in the present study labeled all cells that had been generated within the past 3 weeks^56^; therefore, only a portion of the cells were at the critical stage during which training is effective. We found a trend of an increase in the DCX cell count following training in the control group but not in the noise group. It is likely that the training effect is masked by the other cells that are not at the critical stage. Further, Ki67 staining labeled the cells that were in the phase of proliferation several hours^57^ before the animal was sacrificed; obviously those cells had not yet reached the critical stage for rescue. In our lab, research is being conducted to label proliferated cells using BrdU or EdU before training to verify whether NIHL reduces the rescue effect of training on neurogenesis in the hippocampus. We are also investigating whether and how NIHL impacts the expression of learning-related genes in the hippocampus.

In conclusion, the present report suggested a role of NIHL independent of its oxidative effect on cognitive function and hippocampal neurogenesis. Therefore, NIHL, if appropriately established, can be used as a model of sensorineural hearing loss to study the mechanism through which hearing loss promotes the development of dementia.

## Materials and Methods

A total of 96 CBA/CAJ mice were obtained from the Experimental Animal Center of Shanghai Super-B&K Laboratory Animal Corp. Ltd., Shanghai, China. The subjects in the noise group were exposed to an intense broadband noise at 123 dB SPL for 2 hours at the age of 6 and 8 weeks, while the control subjects underwent sham exposure to the environmental change.

Blood samples were taken from 8 subjects in each group at 0, 1, 7 and 28 day(s) after noise exposure to examine serum CORT level. They were returned to the colony for further evaluation. Twenty-four subjects in the noise group were sacrificed (8 at each of the three time points of 1 day, 1 month and 3 months after noise exposure) for the examination of oxidative stress in the hippocampus, and 8 subjects in the control group were sacrificed at 1 m after the sham exposure for the same evaluation.

Three months after the noise/sham exposure, all animals remaining (32 in each group) were examined for hearing threshold by the frequency specific auditory brainstem response (ABR). In each group, the 32 subjects were further divided into trained (n = 22) and non-trained (n = 10) subgroups. The capabilities of spatial learning and memory were measured in each trained subgroup by means of a Morris water maze. Immediately after the functional test, the hippocampus was harvested for the observation of neurogenesis, and the cochleae were harvested from 12 subjects in the noise group for the evaluation of hair cell loss. All animal procedures were performed in accordance with the guidelines in the ethic protocol approved by the University Committee for Laboratory Animals of Southeast University, China (Permit number: SCXK2011-0003).

### Noise exposure

The animals were unrestrained in a cage 60 cm below the horns of two loudspeakers, a low frequency woofer and a high frequency tweeter. Electrical Gaussian noise was delivered to the speakers after power amplification. The acoustic spectrum of the sound was distributed mainly below 20 kHz, as previously reported^58^. The noise level was adjusted to 123 dB SPL and monitored using a ¼-inch microphone linked to a sound level meter (microphone: 2520, sound level meter: 824, from Larson Davis, Depew, NY, USA).

### ABR test

For the ABR recordings, the animal was anesthetized with pentobarbital (60 mg/kg, i.p.), and body temperature maintained at 37.5–38 °C with a thermostatic heating pad. Three subdermal needle electrodes were used to record the ABRs. The non-inverting electrode was inserted at the vertex, and the reference and grounding electrodes were placed on the two earlobes.

Hardware and software (BioSig and SigGen) from Tucker-Davis Technology (TDT system III, Alachua, FL, USA) were used for stimulus generation and bio-signal acquisition. The stimuli were played through a broadband speaker (MF1 from TDT), which was placed 10 cm in front of the animal’s head. The evoked responses were amplified 20 times and digitized via a pre-amplifier (RA16PA) with a filter between 100–3000 Hz. The responses were averaged 1,000 times. The thresholds were measured across frequencies from 2 to 32 kHz with tone bursts presented at the rate of 21.1/s. At each frequency, the test was performed in a descending sequence from 90 dB SPL in 5-dB steps until the ABR response disappeared.

### Behavioral test

The MWM setting was identical to that described previously^59^,^60^. The pool was filled to a depth of 14 cm (0.5 cm over the platform) with tap water at 22–24 °C. The MWM test consisted of two phases. The first one was a 5-day spatial acquisition phase in which four training trials interrupted by an interval of 10 min were performed each day. An acclimatization session was given on the first day, as described previously^61^. The animal was allowed to swim and search for the hidden platform for 60 s during each trial. The escape latency was defined as the time needed for the animal to locate the platform before it could stay steadily on it for more than 5 sec. The animal was guided to the platform if it could not find the platform within 60 s. The second phase (the probe trial) was performed the next to last day of the first phase with the platform removed. Each mouse was allowed to swim for 60 s in the pool. The number of times the mouse went across the location of the platform was recorded as the index of memory of the platform.

### Oxidative stress

Blood samples were taken from the tail vein repeatedly from 8 subjects in each group at 0, 1, 7 and 28 day(s) after noise exposure to examine serum CORT level. Each sample was placed in a sodium heparin-coated tube, and 50 μl of supernatant plasma was obtained from each sample after centrifugation for testing. The plasma CORT concentration was measured using a Mouse Corticosterone (CORT) ELISA Kit (CSB-E07969 m, CUSABIO, Wuhan, China), strictly following the kit instructions.

Hippocampal tissue was quickly harvested on ice from mice after decapitation and was immersed in PBS of 9 times the tissue volume for ultrasonication. Supernatant was obtained after ultracentrifugation for the tests of three common indicators of oxidative stress. Commercial test kits from Cell Biolabs (San Diego, USA) were used for measuring super oxide dismutase (SOD) activity (Superoxide Dismutase Activity Assay kit STA-340), malondialdehyde (MDA) adducts (ELISA Kit, STA-332) and reactive oxygen/nitrogen species (ROS/RNS) level (*In Vitro* ROS/RNS Assay Kit, STA-347). Total protein concentration was measured before the MDA test using the Pierce® BCA Protein Assay Kit (23225, Thermo, USA). The ROS/RNS level was tested using a ROS/RNS-sensitive fluorescent dye, DCF (2′,7′-dichlorofluorescin), which was generated from the reaction of ROS/RNS with a specific probe: DCFH-DA (dichlorofluorescein diacetate). The compound is hydrolyzed to DCF, which interacts with ROS/RNS to generate fluorescence. The quantity of ROS/RNS was calculated based on the standard obtained using the known ROS/RNS contents.

### Neurogenesis

The animal was deeply anesthetized with pentobarbital (100 mg/kg, i.p.) and fixed with open-chest cardiovascular perfusion of 4% paraformaldehyde in PBS buffer followed by post-fixation in the same fixative at 4 °C for 24 h. The brain tissue block was then immersed in 30% sucrose, dehydrated at 4 °C until it sunk to the bottom, embedded in OCT compound and then frozen in a −80 °C freezer. The frozen block was sliced into 25 μm thick sections using a microtome (Leica Cryostat Microtome 1900, Heidelberger, Germany). One section from every 3 was chosen for further processing.

Doublecortin (DCX) staining was performed using floating sections. The selected sections were permeabilized with 0.1% Triton X-100 in PBS for 30 min, incubated for 30 min in 10% donkey serum in PBS and then incubated in the primary antibody (1:300, goat polyclonal anti-DCX, sc-8066, Santa Cruz, Santa Cruz, CA, USA) overnight at 4 °C. This was followed by treatment with secondary antibodies (1:500, Donkey Anti Goat IgG-H&L Cy3®, ab6949, Abcam, Cambridge, UK) for 1 h at room temperature. Ki67 staining was performed on affixed tissue sections. The selected slices were placed onto lysine-treated glass slides, dried at room temperature, dipped in antigen retrieval solution (mixture of citric acid buffer), and then heated in a microwave oven. The slices were then permeabilized with 0.1% Triton X-100 in PBS for 30 min, incubated for 30 min in 10% goat serum and then incubated in the primary antibody (1:300, Rabbit polyclonal to Ki67, ab66155, Abcam, Cambridge, UK) overnight at 4 °C. This was followed by incubation with secondary antibodies (1:1000, Cy2-AffiniPure Goat Anti-Rabbit IgG-H&L, 111-225-045, Jackson ImmunoResearch, West Grove, PA, USA) for 1 h at room temperature. The numbers of DCX- and Ki67-positive cells were counted under a fluorescence microscope (OLYMPUS BX53, Tokyo, Japan) and multiplied by 3 to yield the total number of DCX/Ki67-positive cells in the whole DG region in each animal brain.

### SDH staining for hair cell count

The mouse was anaesthetized (sodium pentobarbital, 100 mg/kg, i.m.) and decapitated. The otocyst was quickly removed, and the round and oval windows were picked under an anatomical microscope. The cochlea was perfused with the succinate dehydrogenase (SDH) staining solution, which contained 0.2 M succinate dibasic hexahydrate (substrate, Sigma, St. Louis, USA) and 0.1% Tetranitro Blue Tetrazolium Chloride (Nitro-BT, Sigma, St. Louis, USA) in 0.2 M PBS. Then, the cochlea was immersed in the solution for 45 min at 37 °C before being transferred to 4% paraformaldehyde for 24 h at 4 °C and decalcified with 1N HCl. After that, the organ of Corti was dissected and mounted with 80% glycerin in PBS on a glass slide. It was observed under a light microscope, and the missing hair cells were counted to establish a cochleogram^4^.

### Statistics

All statistical analyses were performed using SigmaPlot 12.2 software. The ANOVA tests were performed before the post-hoc tests. All data are presented as the means ± SEM, unless otherwise specified. Significance is accepted at the level of p < 0.05.

## Additional Information

**How to cite this article**: Liu, L. *et al.* Noise induced hearing loss impairs spatial learning/memory and hippocampal neurogenesis in mice. *Sci. Rep.* **6**, 20374; doi: 10.1038/srep20374 (2016).

## References

[b1] Tucci D., Merson M. H. & Wilson B. S. A summary of the literature on global hearing impairment: current status and priorities for action. Otol Neurotol 31, 31–41 (2010). 20050266 10.1097/mao.0b013e3181c0eaec

[b2] Agrawal Y., Platz E. A. & Niparko J. K. Prevalence of hearing loss and differences by demographic characteristics among US adults: data from the National Health and Nutrition Examination Survey, 1999–2004. Arch Intern Med 168, 1522–1530, doi: 10.1001/archinte.168.14.1522 (2008). 18663164

[b3] Cruickshanks K. J. *et al.* Prevalence of hearing loss in older adults in Beaver Dam, Wisconsin. The Epidemiology of Hearing Loss Study. Am J Epidemiol 148, 879–886 (1998). 9801018 10.1093/oxfordjournals.aje.a009713

[b4] Nelson D. I., Nelson R. Y., Concha-Barrientos M. & Fingerhut M. The global burden of occupational noise-induced hearing loss. American journal of industrial medicine 48, 446–458, doi: 10.1002/ajim.20223 (2005). 16299704

[b5] Basner M. *et al.* Auditory and non-auditory effects of noise on health. Lancet 383, 1325–1332, doi: 10.1016/S0140-6736(13)61613-X (2014). 24183105 PMC3988259

[b6] Cheng L., Wang S. H., Chen Q. C. & Liao X. M. Moderate noise induced cognition impairment of mice and its underlying mechanisms. Physiol Behav 104, 981–988, doi: 10.1016/j.physbeh.2011.06.018 (2011). 21726571

[b7] Cui B., Wu M. & She X. Effects of chronic noise exposure on spatial learning and memory of rats in relation to neurotransmitters and NMDAR2B alteration in the hippocampus. Journal of occupational health 51, 152–158 (2009). 19225220 10.1539/joh.l8084

[b8] Jauregui-Huerta F. *et al.* Chronic exposure of juvenile rats to environmental noise impairs hippocampal cell proliferation in adulthood. Noise Health 13, 286–291, doi: 10.4103/1463-1741.82961 (2011). 21768732

[b9] Wright B., Peters E., Ettinger U., Kuipers E. & Kumari V. Understanding noise stress-induced cognitive impairment in healthy adults and its implications for schizophrenia. Noise Health 16, 166–176, doi: 10.4103/1463-1741.134917 (2014). 24953882

[b10] Helfer T. M. *et al.* Noise-induced hearing injury and comorbidities among postdeployment U.S. Army soldiers: April 2003-June 2009. Am J Audiol 20, 33–41, doi: 10.1044/1059-0889(2011/10-0033) (2011). 21474555

[b11] Cave K. M., Cornish E. M. & Chandler D. W. Blast injury of the ear: clinical update from the global war on terror. Military medicine 172, 726–730 (2007). 17691685 10.7205/milmed.172.7.726

[b12] Belanger H. G., Kretzmer T., Yoash-Gantz R., Pickett T. & Tupler L. A. Cognitive sequelae of blast-related versus other mechanisms of brain trauma. J Int Neuropsychol Soc 15, 1–8, doi: 10.1017/S1355617708090036 (2009). 19128523

[b13] Chengzhi C. *et al.* Recovery of chronic noise exposure induced spatial learning and memory deficits in young male Sprague-Dawley rats. Journal of occupational health 53, 157–163 (2011). 21422718 10.1539/joh.l10125

[b14] Hirano Y. *et al.* Effect of unpleasant loud noise on hippocampal activities during picture encoding: an fMRI study. Brain Cogn 61, 280–285, doi: 10.1016/j.bandc.2006.02.003 (2006). 16581168

[b15] Kraus K. S. & Canlon B. Neuronal connectivity and interactions between the auditory and limbic systems. Effects of noise and tinnitus. Hear Res 288, 34–46, doi: 10.1016/j.heares.2012.02.009 (2012). 22440225

[b16] Yu Y. F., Zhai F., Dai C. F. & Hu J. J. The relationship between age-related hearing loss and synaptic changes in the hippocampus of C57BL/6J mice. Exp Gerontol 46, 716–722, doi: 10.1016/j.exger.2011.04.007 (2011). 21586320

[b17] Kraus K. S. *et al.* Noise trauma impairs neurogenesis in the rat hippocampus. Neuroscience 167, 1216–1226, doi: 10.1016/j.neuroscience.2010.02.071S0306-4522(10)00305-2 (2010). 20206235 PMC2952397

[b18] Cui B., Wu M., She X. & Liu H. Impulse noise exposure in rats causes cognitive deficits and changes in hippocampal neurotransmitter signaling and tau phosphorylation. Brain Res 1427, 35–43, doi: S0006-8993(11)01540-X10.1016/j.brainres.2011.08.035 (2011). 22055774 10.1016/j.brainres.2011.08.035

[b19] Xu J., Yu L., Cai R., Zhang J. & Sun X. Early continuous white noise exposure alters auditory spatial sensitivity and expression of GAD65 and GABAA receptor subunits in rat auditory cortex. Cereb Cortex 20, 804–812, doi: 10.1093/cercor/bhp143 (2010). 19620619

[b20] Rabat A., Bouyer J. J., George O., Le Moal M. & Mayo W. Chronic exposure of rats to noise: relationship between long-term memory deficits and slow wave sleep disturbances. Behav Brain Res 171, 303–312, doi: S0166-4328(06)00210-5 10.1016/j.bbr.2006.04.007 (2006). 16716416 10.1016/j.bbr.2006.04.007

[b21] Stansfeld S. A. *et al.* Aircraft and road traffic noise and children’s cognition and health: a cross-national study. Lancet 365, 1942–1949, doi: S0140-6736(05)66660-3 10.1016/S0140-6736(05)66660-3 (2005). 15936421 10.1016/S0140-6736(05)66660-3

[b22] Haines M. M., Stansfeld S. A., Job R. F., Berglund B. & Head J. Chronic aircraft noise exposure, stress responses, mental health and cognitive performance in school children. Psychol Med 31, 265–277 (2001). 11232914 10.1017/s0033291701003282

[b23] Arnsten A. F. & Goldman-Rakic P. S. Noise stress impairs prefrontal cortical cognitive function in monkeys: evidence for a hyperdopaminergic mechanism. Arch Gen Psychiatry 55, 362–368 (1998). 9554432 10.1001/archpsyc.55.4.362

[b24] Manikandan S. *et al.* Effects of chronic noise stress on spatial memory of rats in relation to neuronal dendritic alteration and free radical-imbalance in hippocampus and medial prefrontal cortex. Neurosci Lett 399, 17–22, doi: 10.1016/j.neulet.2006.01.037 (2006). 16481110

[b25] Schoenfeld T. J. & Gould E. Stress, stress hormones, and adult neurogenesis. Exp Neurol 233, 12–21, doi: S0014-4886(11)00022-7 10.1016/j.expneurol.2011.01.008 (2012). 21281629 10.1016/j.expneurol.2011.01.008 PMC3715962

[b26] Li W. Z. *et al.* Glucocorticoids increase impairments in learning and memory due to elevated amyloid precursor protein expression and neuronal apoptosis in 12-month old mice. Eur J Pharmacol 628, 108–115, doi: 10.1016/j.ejphar.2009.11.045 (2010). 19948164

[b27] Schoenfeld T. J. & Gould E. Differential effects of stress and glucocorticoids on adult neurogenesis. Curr Top Behav Neurosci 15, 139–164, doi: 10.1007/7854_2012_233 (2013). 23670817

[b28] Wang Q. *et al.* Glucocorticoid receptor protein expression in human hippocampus; stability with age. Neurobiol Aging 34, 1662–1673, doi: 10.1016/j.neurobiolaging.2012.11.019 (2013). 23290588

[b29] Lucassen P. J. *et al.* Neuropathology of stress. Acta Neuropathol 127, 109–135, doi: 10.1007/s00401-013-1223-5 (2014). 24318124 PMC3889685

[b30] Lucassen P. J. *et al.* Regulation of Adult Neurogenesis and Plasticity by (Early) Stress, Glucocorticoids, and Inflammation. Cold Spring Harb Perspect Biol 7, doi: 10.1101/cshperspect.a021303 (2015). PMC4563706 26330520

[b31] Hunter R. G., Gagnidze K., McEwen B. S. & Pfaff D. W. Stress and the dynamic genome: Steroids, epigenetics, and the transposome. Proc Natl Acad Sci USA 112, 6828–6833, doi: 10.1073/pnas.1411260111 (2015). 25385609 PMC4460487

[b32] Lucassen P. J. *et al.* Stress, depression and hippocampal apoptosis. CNS Neurol Disord Drug Targets 5, 531–546 (2006). 17073656 10.2174/187152706778559273

[b33] Snyder J. S., Soumier A., Brewer M., Pickel J. & Cameron H. A. Adult hippocampal neurogenesis buffers stress responses and depressive behaviour. Nature 476, 458–461, doi: 10.1038/nature10287 (2011). 21814201 PMC3162077

[b34] Opendak M. & Gould E. New neurons maintain efficient stress recovery. Cell Stem Cell 9, 287–288, doi: 10.1016/j.stem.2011.09.003 (2011). 21982225

[b35] Samson J. *et al.* Noise-induced time-dependent changes in oxidative stress in the mouse cochlea and attenuation by D-methionine. Neuroscience 152, 146–150, doi: 10.1016/j.neuroscience.2007.11.015 (2008). 18234425

[b36] Sarah Hayes S. M., Brian Allman & Richard Salvi. Stress Hormone Levels and Hippocampal Neurogenesis Following Acoustic Trauma in *The 34th Annual Midwinter meeting of Association For Research in Otolaryngology. Baltimore, USA.* Association For Research in Otolaryngology (2011).

[b37] Zheng Y., Hamilton E., Begum S., Smith P. F. & Darlington C. L. The effects of acoustic trauma that can cause tinnitus on spatial performance in rats. Neuroscience 186, 48–56, doi: 10.1016/j.neuroscience.2011.04.052 (2011). 21549180

[b38] Lin F. R. *et al.* Hearing loss and incident dementia. Arch Neurol 68, 214–220, doi: 68/2/214 10.1001/archneurol.2010.362 (2011). 21320988 10.1001/archneurol.2010.362 PMC3277836

[b39] Lin F. R. *et al.* Hearing loss and cognition in the Baltimore Longitudinal Study of Aging. Neuropsychology 25, 763–770, doi: 2011-13415-001 10.1037/a0024238 (2011). 21728425 10.1037/a0024238 PMC3193888

[b40] Lin F. R. Hearing loss and cognition among older adults in the United States. J Gerontol A Biol Sci Med Sci 66, 1131–1136, doi: 10.1093/gerona/glr115 glr115 (2011). 21768501 PMC3172566

[b41] Lin F. R. *et al.* Hearing Loss and Cognitive Decline in Older Adults. JAMA Intern Med. 1–7, doi: 1558452 10.1001/jamainternmed.2013.1868 (2013). 10.1001/jamainternmed.2013.1868 PMC3869227 23337978

[b42] Moxon K. A. *et al.* Multiple single units and population responses during inhibitory gating of hippocampal auditory response in freely-moving rats. Brain Res 825, 75–85 (1999). 10216175 10.1016/s0006-8993(99)01187-7

[b43] O’Mara S. The subiculum: what it does, what it might do, and what neuroanatomy has yet to tell us. J Anat 207, 271–282, doi: 10.1111/j.1469-7580.2005.00446.x (2005). 16185252 PMC1571536

[b44] Shi L. *et al.* Ribbon synapse plasticity in the cochleae of Guinea pigs after noise-induced silent damage. PLoS One 8, e81566, doi: 10.1371/journal.pone.0081566 PONE-D-13-30022 (2013). 24349090 PMC3857186

[b45] Liu L. *et al.* Silent damage of noise on cochlear afferent innervation in guinea pigs and the impact on temporal processing. PLoS One 7, e49550, doi: 10.1371/journal.pone.0049550 PONE-D-12-26008 (2012). 23185359 PMC3504112

[b46] Kujawa S. G. & Liberman M. C. Adding insult to injury: cochlear nerve degeneration after “temporary” noise-induced hearing loss. J Neurosci 29, 14077–14085, doi: 29/45/14077 10.1523/JNEUROSCI.2845-09.2009 (2009). 19906956 10.1523/JNEUROSCI.2845-09.2009 PMC2812055

[b47] Kim H. *et al.* Influence of prenatal noise and music on the spatial memory and neurogenesis in the hippocampus of developing rats. Brain Dev 28, 109–114, doi: 10.1016/j.braindev.2005.05.008 (2006). 16181757

[b48] Curlik D. M. 2nd, Maeng L. Y., Agarwal P. R. & Shors T. J. Physical skill training increases the number of surviving new cells in the adult hippocampus. PLoS One 8, e55850, doi: 10.1371/journal.pone.0055850 PONE-D-12-21761 (2013). 23437067 PMC3577803

[b49] Xu Z. *et al.* Working memory task decreases the survival of newly born neurons in hippocampus. Neurobiol Learn Mem 95, 239–247, doi: S1074-7427(10)00199-1 10.1016/j.nlm.2010.11.013 (2011). 21111839 10.1016/j.nlm.2010.11.013

[b50] Anderson M. L., Sisti H. M., Curlik D. M. 2nd & Shors T. J. Associative learning increases adult neurogenesis during a critical period. Eur J Neurosci 33, 175–181, doi: 10.1111/j.1460-9568.2010.07486.x (2011). 21143670 PMC3057912

[b51] Cameron H. A. & McKay R. D. Adult neurogenesis produces a large pool of new granule cells in the dentate gyrus. J Comp Neurol 435, 406–417 (2001). 11406822 10.1002/cne.1040

[b52] Attardo A. *et al.* Tis21 expression marks not only populations of neurogenic precursor cells but also new postmitotic neurons in adult hippocampal neurogenesis. Cerebral cortex 20, 304–314, doi: 10.1093/cercor/bhp100 (2010). 19482889 PMC2803732

[b53] Veyrac A. *et al.* Zif268/egr1 gene controls the selection, maturation and functional integration of adult hippocampal newborn neurons by learning. Proc Natl Acad Sci USA 110, 7062-7067, doi: 1220558110 10.1073/pnas.1220558110 (2013). 23569253 10.1073/pnas.1220558110 PMC3637756

[b54] Epp J. R., Chow C. & Galea L. A. Hippocampus-dependent learning influences hippocampal neurogenesis. Front Neurosci 7, 57, doi: 10.3389/fnins.2013.00057 (2013). 23596385 PMC3627134

[b55] Gould E., Beylin A., Tanapat P., Reeves A. & Shors T. J. Learning enhances adult neurogenesis in the hippocampal formation. Nat Neurosci 2, 260–265, doi: 10.1038/6365 (1999). 10195219

[b56] Kohler S. J., Williams N. I., Stanton G. B., Cameron J. L. & Greenough W. T. Maturation time of new granule cells in the dentate gyrus of adult macaque monkeys exceeds six months. Proc Natl Acad Sci USA 108, 10326–10331, doi: 10.1073/pnas.1017099108 (2011). 21646517 PMC3121825

[b57] Bruno S. & Darzynkiewicz Z. Cell cycle dependent expression and stability of the nuclear protein detected by Ki-67 antibody in HL-60 cells. Cell proliferation 25, 31–40 (1992). 1540682 10.1111/j.1365-2184.1992.tb01435.x

[b58] Wang J. *et al.* Overexpression of X-linked inhibitor of apoptosis protein protects against noise-induced hearing loss in mice. Gene Ther 18, 560–568, doi: gt2010172 10.1038/gt.2010.172 (2011). 21228883 10.1038/gt.2010.172

[b59] Mendez-Couz M., Conejo N. M., Vallejo G. & Arias J. L. Spatial memory extinction: A c-Fos protein mapping study. Behav Brain Res 260, 101–110, doi: 10.1016/j.bbr.2013.11.032 S0166-4328(13)00719-5 (2014). 24315832

[b60] Bonaccorsi J. *et al.* System consolidation of spatial memories in mice: effects of enriched environment. Neural Plast 2013, 956312, doi: 10.1155/2013/956312 (2013). 23936679 PMC3723323

[b61] Vorhees C. V. & Williams M. T. Morris water maze: procedures for assessing spatial and related forms of learning and memory. Nat Protoc 1, 848–858, doi: nprot.2006.116 10.1038/nprot.2006.116 (2006). 17406317 10.1038/nprot.2006.116 PMC2895266

